# Molecular Storage of Ozone in a Clathrate Hydrate: An Attempt at Preserving Ozone at High Concentrations

**DOI:** 10.1371/journal.pone.0048563

**Published:** 2012-11-05

**Authors:** Takahiro Nakajima, Taisuke Kudo, Ryo Ohmura, Satoshi Takeya, Yasuhiko H. Mori

**Affiliations:** 1 Department of Mechanical Engineering, Keio University, Yokohama, Japan; 2 Research Institute of Instrumentation Frontier, National Institute of Advanced Industrial Science and Technology (AIST), Tsukuba, Japan; Harbin Institute of Technology, China

## Abstract

This paper reports an experimental study of the formation of a mixed O_3_+ O_2_+ CO_2_ hydrate and its frozen storage under atmospheric pressure, which aimed to establish a hydrate-based technology for preserving ozone (O_3_), a chemically unstable substance, for various industrial, medical and consumer uses. By improving the experimental technique that we recently devised for forming an O_3_+ O_2_+ CO_2_ hydrate, we succeeded in significantly increasing the fraction of ozone contained in the hydrate. For a hydrate formed at a system pressure of 3.0 MPa, the mass fraction of ozone was initially about 0.9%; and even after a 20-day storage at −25°C and atmospheric pressure, it was still about 0.6%. These results support the prospect of establishing an economical, safe, and easy-to-handle ozone-preservation technology of practical use.

## Introduction

Ozone (O_3_) is known as a powerful oxidant and, due to this nature, it is widely used for, for example, the decontamination of air and water, the sterilization of perishables, the disinfection of medical instruments, and the cleaning or surface-conditioning processes in the semiconductor industry. However, it is neither very easy nor economical to use ozone in consumer applications such as sanitizing foods and drinking water, removing pesticide residues from fruits and vegetables, treating water in aquariums for suppressing bacteria growth, etc. This is because, to artificially generate ozone, we need a high-voltage electric device such as a corona discharger or a cold plasma generator and, once generated, ozone in the gaseous state rapidly decomposes to oxygen (O_2_). Thus, it is generally believed that ozone can neither be stored nor transported and must be produced on site. For the limited consumer use of ozone, ozonated water (liquid water in which ozone is physically dissolved) and ozonated ice (water ice holding microbubbles of an ozone-containing gas) are commercially available. However, the ozone concentration in such ozonated water or ice is generally on the order of 1 or 10 ppm even in its fresh state, and rapidly decays with time. The above state of affairs seriously restricts the situations allowing the use of ozone. If we find a convenient means for transporting ozone from its production site to any place where ozone is needed, the utility of ozone will be significantly expanded.

Clathrate hydrates (abbreviated hydrates) are crystalline solid compounds each composed of host water molecules hydrogen-bonded into a structure of interlinked cages. Unless the given pressure is extremely high (typically on the order of gigapascals), each cage contains at most one guest molecule of a substance other than water [1]. That is, the guest molecules in a hydrate are isolated by the cage walls due to van der Waals forces and thereby prevented, in general, from mutual interactions. This indicates that hydrates have a high potential of storing chemically unstable substances, such as ozone, in the form of encaged guest molecules.

The idea of storing ozone in a hydrate was first presented by McTurk and Waller [2], [3] in 1964. They reported the formation of an ozone-containing hydrate in an experimental system containing pure ozone, carbon tetrachloride (CCl_4_) and water, and, based on their X-ray diffraction measurement, indicated that this hydrate was a double O_3_+ CCl_4_ hydrate in structure II. However, they provided neither any quantitative evaluation of the ozone content nor any experimental evidence for actual ozone preservation in their hydrate.

The pure ozone used by McTurk and Waller [2], [3] is not easily available. Besides, it is explosive and very difficult to handle in practice. Carbon tetrachloride is effective as a *help guest* for lowering the pressure required for hydrate formation, though it is toxic and may be unsuitable for some applications. An attempt at forming a hydrate from a dilute ozone-containing gas (a mixture of ∼5% O_3_ and ∼95% O_2_ generated from a commercial ozone generator) in the absence of any help guest was reported by Masaoka et al. [4]. They formed a hydrate at a pressure of 13 MPa and a temperature of −25°C, and determined the ozone content of the hydrate to be 2.3 g/L (≈ 0.2% in mass fraction). They also performed a storage test of the hydrate at the same pressure−temperature conditions as those in the formation process, i.e., 13 MPa and −25°C, and observed only a slight decrease in the ozone content of the hydrate during 10-days storage after its formation.

More recently, Muromachi et al. [5] formed a hydrate from a ozone + oxygen gas mixture (∼8% in mole fraction of ozone) and carbon tetrachloride or xenon (Xe) at a pressure of 0.25 or 0.35 MPa and a temperature of 0.1°C. They showed that, if cooled to −20°C under an aerated atmospheric-pressure condition, the O_3_+ O_2_+ CCl_4_ and O_3_+ O_2_+ Xe hydrates could preserve ozone for more than 20 days at mass fractions around 0.2% and 0.1%, respectively. Subsequently, Muromachi et al. [6] performed phase-equilibrium measurements for the O_3_+ O_2_+ CCl_4_ and O_3_+ O_2_+ CH_3_CCl_2_F hydrates.

For practical applications of ozone-containing hydrates, the use of a toxic or very expensive substance as the help guest should be avoided. On the other hand, the hydrates can be desirably formed at moderate pressures and preserved at a moderately cooled atmospheric-pressure condition. In order to satisfy these requirements, Nakajima et al. [7] selected carbon dioxide (CO_2_) as the help guest, and formed an O_3_+ O_2_+ CO_2_ hydrate from an O_3_+ O_2_ gas mixture (10−12% in O_3_ mole fraction) blended with pure CO_2_ in a molar ratio of 1∶7 at a pressure of 1.9 MPa and a temperature of 0.1°C. They performed preservation tests with the O_3_+ O_2_+ CO_2_ hydrate at different storage temperatures from −5°C to −30°C under an aerated atmospheric-pressure condition. The results of these tests showed that, for ozone preservation over 20 days at a mass fraction around 0.1%, the storage temperature should be −25°C or lower. This inferiority in ozone-preserving function of the O_3_+ O_2_+ CO_2_ hydrate in comparison with the O_3_+ O_2_+ CCl_4_ hydrate [5] is, as demonstrated by a relevant phase-equilibrium study [8], essentially due to the higher phase-equilibrium pressure for the former than the latter at any given temperature and hence inevitable. Despite such a thermodynamic disadvantage of the O_3_+ O_2_+ CO_2_ hydrate as compared to the O_3_+ O_2_+ CCl_4_ hydrate, the former represents a good compromise between the ozone preservability versus the biological safety and the economy in system operation, and is possibly the best selection as the ozone storage medium for practical use. Moreover, we can expect that the gas mixture released from an O_3_+ O_2_+ CO_2_ hydrate will have some synergetic effect of the ozone and carbon dioxide for sterilizing foods [9] and will possibly be more suitable for food-industrial applications than the O_3_+ O_2_ or O_3_+ air gas mixtures directly generated from commercial ozone generators.

This study is an extension of the first O_3_+ O_2_+ CO_2_ hydrate study by Nakajima et al. [7] discussed above. The major objectives of this study were (a) to generate O_3_+ O_2_+ CO_2_ hydrates having higher ozone fractions, and (b) to perform ozone preservation tests with these hydrates in order to examine their practical utility. The study was successful regarding both of these objectives. We confirmed that, with simple modifications of the hydrate-forming procedure, the initial ozone content of a hydrate can be multiplied severalfold as compared to that previously observed [7], and that more than half of such a high ozone content remains after a 20-day hydrate storage under an aerated atmospheric-pressure condition at a temperature of −25°C.

## Experimental Section

The general experimental scheme used in this study was the same as that used in our previous study [7] that first dealt with an O_3_+ O_2_+ CO_2_ hydrate. However, some core portions of the hydrate-forming apparatus were modified this time in order to increase the water-to-hydrate conversation ratio (i.e., to reduce the fraction of ice in the hydrate + ice solid mixtures for use in ozone preservation tests) and to allow pressurizing the O_3_+ O_2_ gas mixture released from an ozone generator before mixing it with CO_2_ gas. Details of the materials, equipment and procedure used in this study are described below.

### Materials

The raw materials used for forming the O_3_+ O_2_+ CO_2_ hydrates were oxygen certified to the purity of 99.9% (volume basis) and carbon dioxide certified to the purity of 99.995% (volume basis) by their supplier (Japan Fine Products Corp., Kawasaki, Kanagawa Prefecture, Japan), and water deionized and distilled in our laboratory. Oxygen was used for generating an O_3_+ O_2_ gas mixture (>11% in mole fraction of O_3_) with the aid of a dielectric-barrier-discharge-based ozone generator (ED-OGS-HP1, EcoDesign Co., Ltd., Saitama Prefecture, Japan).

### Apparatus

**Figure 1:**
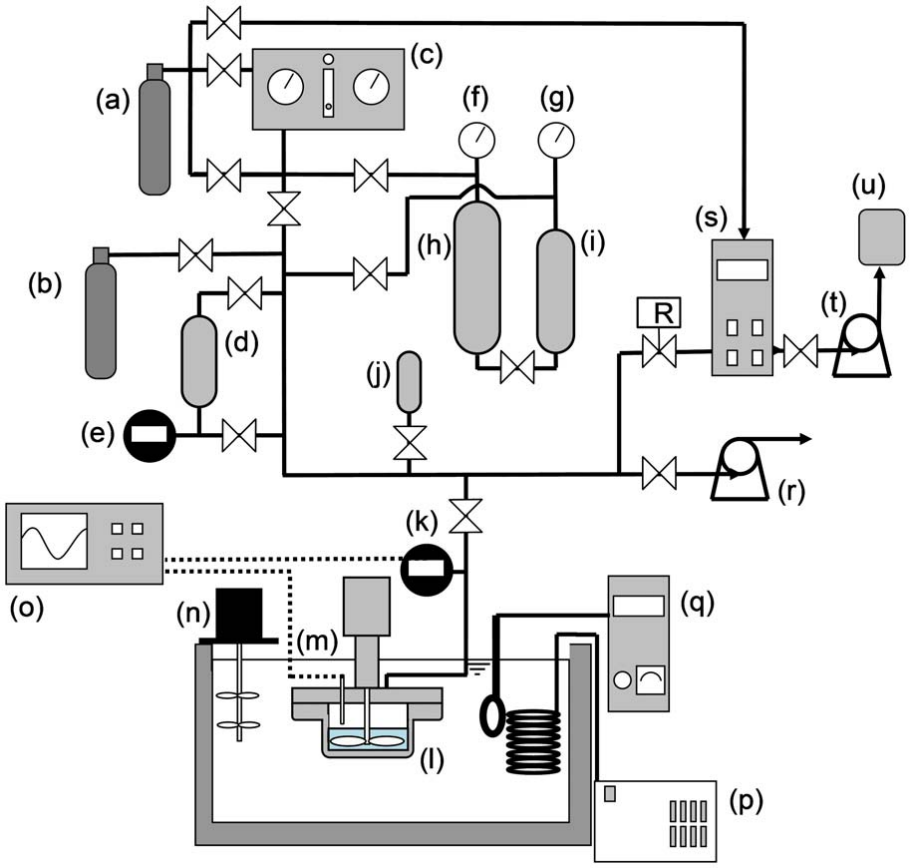
Schematic illustration of the experimental setup for forming the O_3_+ O_2_+ CO_2_ hydrates. This setup consists of (a) an oxygen cylinder, (b) a carbon-dioxide cylinder, (c) an ozone generator, (d) a gas-mixing chamber, (e) a pressure gauge, (f) and (g) pressure gauges, (h) and (i) gas-pressurizing chambers, (j) a gas-sampling chamber, (k) a pressure gauge, (l) a hydrate-forming reactor, (m) a Pt-wire resistance thermometer, (n) a stirrer, (o) a data logger, (p) an immersion cooler, (q) a PID-controlled heater, (r) a vacuum pump, (s) an ozone monitor, (t) a vacuum pump, and (u) an ozone decomposer.

The experimental setup used to form the hydrates is schematically illustrated in Fig. 1. By comparing this figure to Fig. S1 in our previous paper [7], one may realize how the setup used this time had been modified from its predecessor. The hydrate-forming reactor [indicated as (l) in Fig. 1] was completely renewed. It was a pan-type stainless-steel vessel with a 65-mm inside diameter and 100-cm^3^ inside volume. An impeller-blade stirrer magnetically connected to the drive shaft of an external, variable-speed, ac motor was inserted into this reactor in order to provide its contents with stronger mixing than a magnetic stirrer inside a tall cylindrical reactor used in the previous setup [7] did. As before, the reactor was immersed in a temperature-controlled bath containing an aqueous ethylene-glycol solution.

Another modification in the experimental setup was the installation of two gas-pressurizing chambers, which are indicated as (h) and (i) in Fig. 1. They were stainless-steel cylinders with capacities of 3785 cm^3^ and 2250 cm^3^, respectively. The smaller chamber (i) was used to store the O_3_+ O_2_ gas mixture supplied from the ozone generator (c) at a pressure up to about 0.3 MPa, while the larger one (h) was initially charged with water. By injecting oxygen gas supplied from the external high-pressure cylinder (a) into the larger chamber, water could be displaced from the larger chamber to the smaller chamber, thereby increasing the pressure of the O_3_+ O_2_ gas mixture to the prescribed level.

### Procedure

The procedure of forming an O_3_+ O_2_+ CO_2_ hydrate using the renewed setup (Fig. 1) is as described below. First, the reactor (l) was charged with ∼30 g of water and immersed in a bath of an aqueous ethylene-glycol solution temperature-controlled at 0.1°C. The reactor, the gas-pressurizing chambers (h) and (i), and the gas-mixing chamber (d) were then flushed at least five times with pure oxygen gas, then evacuated. After confirming that the mole fraction of ozone in the gas mixture released from the ozone generator (c) was in the range of 10−12%, the smaller gas-pressurizing chamber (i) and the gas-mixing chamber (d) were charged with this mixture up to a pressure of 0.3 MPa. Oxygen gas from the high-pressure cylinder (a) was then injected into the larger gas-pressurizing chamber (h) to make the water stored in it flow into the smaller chamber (i) and thereby to make the O_3_+ O_2_ gas mixture flow out of the latter chamber into the gas-mixing chamber (d), until the pressure inside the gas-mixing chamber increased to the prescribed level. When the pressure inside the gas-mixing chamber did not sufficiently increase at this stage, the above serial operations beginning with the charging of the smaller gas-pressurizing chamber with a fresh O_3_+ O_2_ gas mixture was repeated until the pressure was raised to the prescribed level. Subsequently, CO_2_ gas was supplied to the gas-mixing chamber until the pressure inside increased to 3 or 4 MPa. The O_3_+ O_2_+ CO_2_ gas mixture thus prepared was supplied to the hydrate-forming reactor (l) until the pressure inside the reactor increased to the prescribed level (2.0, 2.5 or 3.0 MPa). A series of intermittent batch operations for forming a hydrate was then started by turning on the stirrer in the reactor. For preventing the system pressure from significantly decreasing from the prescribed level and for minimizing the change in composition of the gas mixture inside the reactor, each batch operation was not allowed to continue for a long period but interrupted by a gas-exchange operation for replacing the residual gas mixture inside the reactor by a fresh gas mixture newly prepared in, and supplied from, the gas-mixing chamber (d). Such a change in the batch and gas-exchange operations was repeated several times until no decrease in system pressure was detected during each batch operation and hence we judged that the hydrate formation had already ceased. The reactor was then cooled to −15°C to freeze the contents of the reactor except for the residual gas mixture. The gas mixture was then discharged into a 50-cm^3^ gas-sampling chamber (j) for its compositional analysis using a gas chromatograph (Agilent 3000 Micro Gas Chromatograph). After removing the reactor from the bath and dipping it into a liquid-nitrogen pool, the formed hydrate was removed from the reactor and crushed into particles with a 5−7 mm linear dimension. A small portion (∼1−2 g in mass) of the hydrate was sampled for an iodometric measurement for determining its initial ozone content. The rest of the hydrate was stored in a Pyrex test tube, if it was to be used for a subsequent preservation test.

The procedure of the ozone preservation tests performed in this study was completely the same as that employed in our previous studies [5. 7], hence its description is omitted here. The technique used in the PXRD measurement with some hydrate sample was also the same as that described elsewhere [7].

## Results and Discussion

Before presenting the results of a series of ozone preservation tests performed in this study, we need to specify the actual contents of what we have called “hydrates” and “ozone fractions” in the preceding sections in relation to the previous hydrate-preservation studies [4], [5], [7]. Because liquid water used for forming hydrates in any of these studies could not be completely converted to a hydrate, each preservation-test specimen prepared by cooling the formed hydrate, together with residual water, to a test temperature below 0°C must have been a mixture of the hydrate and water ice. Inevitably, the “ozone faction” measured by some macroscopic means (e.g., the iodometric technique [5], [7]) is not an intrinsic ozone fraction of the hydrate but an *effective* fraction defined as the ratio of the mass of ozone contained in a given hydrate + ice mixture to that of the mixture itself. This means that an increase in such an effective ozone fraction, 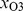, may be achieved either by (i) decreasing the ice fraction in the hydrate + ice mixtures for storing ozone or by (ii) increasing the true ozone fraction in the hydrate. As described in the Experimental Section, we attempted at realizing both of these means in order to significantly increase 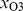 as compared to its magnitude (∼0.1%) observed in the previous study of this series [7]. The former means was realized by intensifying the stirring of the gas/liquid contents in the hydrate-forming rector, while the latter was carried out by varying the system pressure as well as the composition of the feed gas supplied to the reactor during each hydrate-forming operation. Varying the feed-gas composition was made by controlling the ratio of CO_2_ addition to the O_3_+ O_2_ mixture generated from an ozone generator at a nearly fixed O_3_ fraction (10−12%).

**Figure 2:**
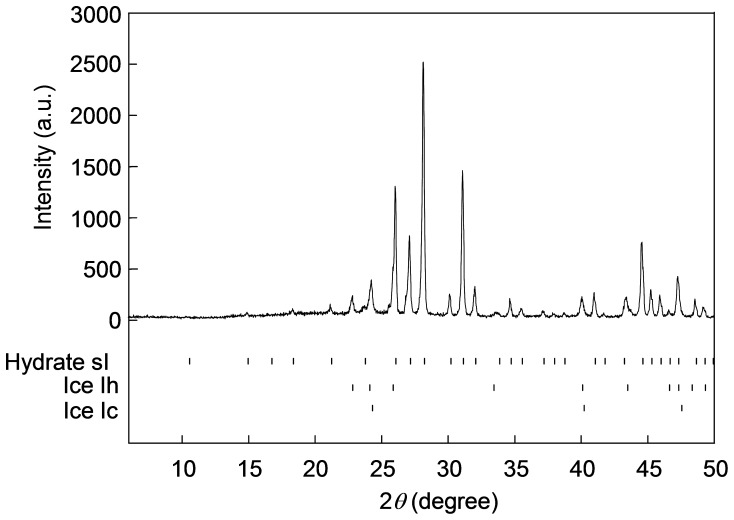
PXRD profile of an O_3_+ O2+ CO_2_ hydrate at 98 K. The solid curve shows the intensities observed using Cu−Kα radiation. The top row of tick marks represent the calculated peak positions for the structure I hydrate, and the lower two rows represent those for the hexagonal ice Ih and cubic ice Ic, respectively. The hydrate sample (accompanied by ice crystals) used in this PXRD measurement was formed from a mixture of O_3_+ O2 and CO_2_ in a nearly 2∶ 8 molar ratio at the condition of *p* = 2.0 MPa and *T* = 0.1°C.

Figure 2 shows a powder X-ray diffraction (PXRD) pattern (measured at 98 K) of an O_3_+ O_2_+ CO_2_ hydrate formed from a mixture of O_3_+ O_2_ and CO_2_ in a nearly 2∶8 molar ratio at a system pressure *p* of 2.0 MPa and a temperature *T* of 0.1°C. This pattern indicates that the hydrate sample used here was a mixture of a hydrate in structure I (sI) with the lattice constant of 11.8294(4) Å and water ice in two different crystal forms, i.e., hexagonal ice, Ih, and cubic ice, Ic. The mass fraction of the hydrate was estimated to be 0.89, which was significantly higher than the corresponding estimate (∼0.3) for the O_3_+ O_2_+ CO_2_ hydrate samples formed in the previous study [7].

**Figure 3:**
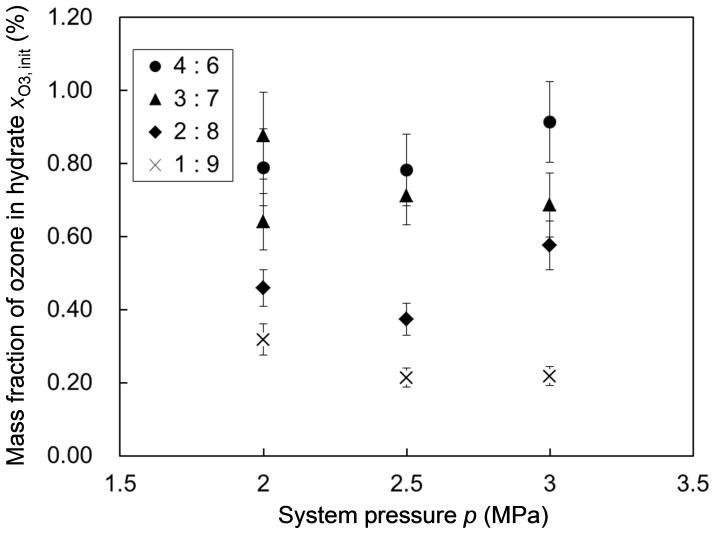
The initial ozone fraction in the formed hydrate versus the system pressure. The legend inserted in the graph indicates the O_3_+ O_2_ versus CO_2_ molar ratio in the feed gas used for each operation. Each data point represents the arithmetic mean of the three 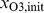 values obtained for the different hydrate samples. The error bar for each data point represents the uncertainty of the ozone-fraction measurement by iodometry.

**Figure 4:**
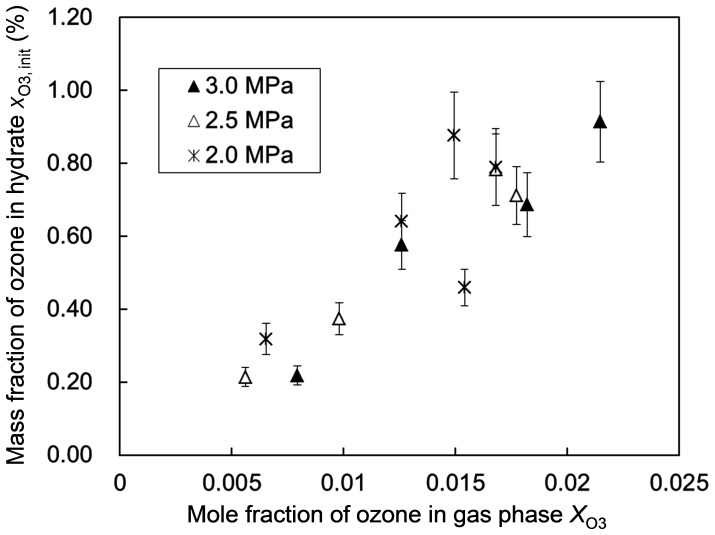
The initial ozone fraction in the formed hydrate versus the gas-phase composition. The mole fraction of ozone, *X*_O3_, shown here is for the gas phase inside the reactor when the hydrate formation ceased. The legend inserted in the graph indicates the system pressure *p* during each hydrate-forming operation. The error bar for each data point represents the uncertainty of the ozone-fraction measurement by iodometry.

We performed hydrate-forming experiments at three different system pressures (2.0, 2.5 and 3.0 MPa) and four different O_3_+ O_2_ versus CO_2_ molar ratios (1∶ 9, 2∶ 8, 3∶7 and 4∶ 6, each accompanied by slight run-to-run scatter) in the feed gas. The hydrate formed in each experiment was subjected to an iodometric measurement to determine its ozone content, i.e., the initial 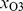 value for the hydrate which we denote 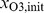 hereafter. Figures 3 and 4 show the variations in 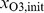 depending on the system pressure *p* for each hydrate-forming operation and the feed-gas composition, respectively, in which the feed-gas composition is represented by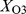, the mole fraction of ozone in the gas phase in contact with the hydrate at the end of each hydrate-forming operation (consult Tables S1 and S2 and Fig. S1 in Supporting Information S1 for the complete sets of 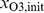 and 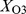 data and the graphical plots of the 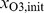 data). We note that 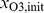 shows no systematic dependence on the system pressure *p* (Fig. 3) but a quasi-linear dependence on 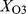 (Fig. 4). This fact indicates that 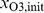 was primarily controlled by the competitive fractional filling of the hydrate cages by O_3_, O_2_ and CO_2_ molecules and that most of the hydrate cages were occupied by some of these guest molecules even at the lowest system pressure, *p* = 2.0 MPa, prescribed in the present experiments. Our estimation of the cage occupancies by O_3_, O_2_ and CO_2_ molecules is described in Supporting Information S2. Consult Table S3 and Fig. S2 in Supporting Information S2 for the estimated occupancy values.

**Figure 5:**
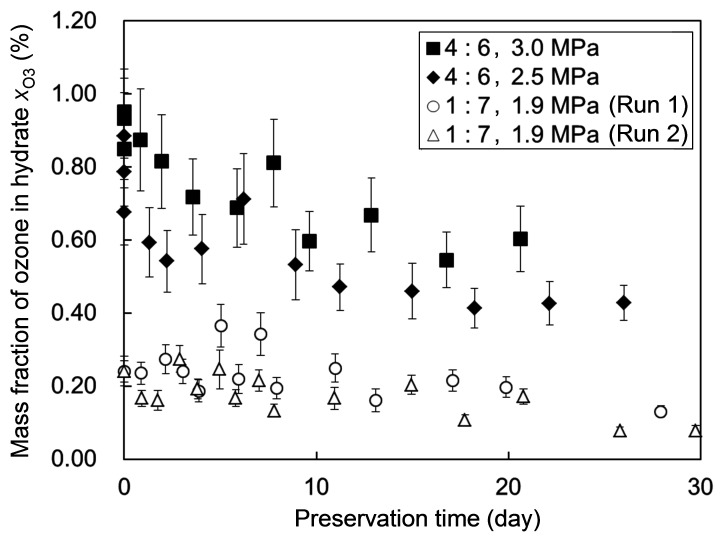
Results of the ozone preservation tests. This graph shows the time evolution of ozone fraction (mass basis) in each O_3_+ O_2_+ CO_2_ hydrate stored under an aerated atmospheric-pressure (0.101 MPa) condition temperature-controlled at −25°C. The comparison of the ozone preservation test data obtained in this study (marked by closed symbols) and those from a previous study [7] (marked by open symbols) are compared. The legend inserted in the graph indicates the O_3_+ O_2_ versus CO_2_ molar ratio in the feed gas and the system pressure *p* for each hydrate-forming operation. The error bar for each data point represents the uncertainty of the ozone-fraction measurement by iodometry.

For the hydrates formed at the two higher system pressures, *p* = 2.5 and 3.0 MPa, ozone preservation tests were performed following the procedure employed in the previous relevant studies [5], [7]. The hydrates were stored in an aerated atmospheric-pressure (0.101 MPa) condition temperature-controlled at −25°C. Figure 5 shows the results of these tests, i.e., two *x*_O3_-data sets each obtained by continually sampling the stored hydrate for iodometric measurements during a period extending to 20−26 days after the formation of the hydrate. In addition, Fig. 5 shows, for comparison, two *x*_O3_-data sets previously obtained with hydrates formed at a lower system pressure and a lower (O_3_+ O_2_)-to-CO_2_ ratio. Obviously, the data obtained in the present preservation tests show much higher 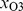 values than the previous data through the entire hydrate-storage period. The former exhibited 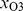 values of 0.4−0.6% at stages of ∼20-days storage, which is a few times higher than the 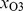 values exhibited by the latter at the same stages. The relatively sharp decrease in 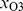 observed in the present tests, particularly during the initial several days of storage, is presumably ascribable to the higher O_3_+ O_2_ fractions (or the lower CO_2_ fractions) in the hydrates used in the tests as compared to the hydrates used in the previous study [7]. Because the thermodynamic equilibrium condition shifts toward a higher pressure or lower temperature with an increase in the O_3_+ O_2_ fraction, or a decrease in the CO_2_ fraction, in the hydrate [8], we can reasonably assume that the hydrates used in the present preservation tests suffered higher thermodynamic driving forces for their dissociation than those used in the previous study [7] under the same storage condition. In addition, the lower ice fraction mixed with the hydrates used in the present tests possibly weakened the ice-barrier effect for suppressing the hydrate dissociation. As recognized in Fig. 5, the present test data show an asymptotic decrease in 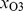 with time, with an apparent half-life period of 20−25 days. This period is considered to be long enough to allow the practical use of ozone-containing hydrates for most industrial, medical and consumer needs.

### Conclusions

This study demonstrated that ozone can be stored up to a mass fraction of ∼0.9% in a structure-I hydrate (containing water ice by ∼10%) formed from a ternary (ozone + oxygen + carbon dioxide) gas mixture containing ozone up to a mole fraction of ∼2%. This is the highest record of an ozone fraction in artificially formed hydrates ever reported in the literature. Such an ozone fraction in a formed hydrate may be further increased by increasing the ozone fraction in the feed gas at the cost of the increasing risk of its explosion [10]. The magnitude of the in-hydrate ozone fraction that we achieved in this study seems to be a good compromise between the demand for increasing the ozone content of a formed hydrate and the need for securing operational safety of the hydrate-forming process using an ozone-containing gas mixture as the feed gas.

Besides the magnitude of the ozone fraction in a freshly formed hydrate, the preservability of ozone encaged in the hydrate is of practical importance. The preservation tests performed in this study revealed that the in-hydrate ozone fraction asymptotically decreases with time from its initial value, about 0.8−0.9%, but still remains at about 0.4−0.6% after a 20-day hydrate storage in an aerated atmospheric-pressure condition cooled at −25°C. This finding strongly suggests the practical utility of mixed ozone + oxygen + carbon dioxide hydrates for the industrial, medical and consumer uses of ozone.

## Supporting Information

Supporting Information S1 **This document contains Table S1, Table S2 and Figure S1.**
(PDF)

Supporting Information S2 **This document contains Table S3 and Figure S2.**
(PDF)
