# Supplementary material for: Molecular Storage of Ozone in a Clathrate Hydrate: An Attempt at Preserving Ozone at High Concentrations
Source: PLoS One. 2012 Nov 5;7(11):e48563. doi: 10.1371/journal.pone.0048563 (PMC3489668; doi:10.1371/journal.pone.0048563)
Supplement: Supporting Information S1 — This document contains Table S1, Table S2 and Figure S1. (PDF) [file pone.0048563.s001.pdf]

Molecular Storage of Ozone in a Clathrate Hydrate: An Attempt at Preserving Ozone at High Concentrations

Takahiro Nakajima, Taisuke Kudo, Ryo Ohmura, Satoshi Takeya, and Yasuhiko H. Mori

---

Supporting Information S1:

Experimental data of hydrate and gas-phase compositions

The experimental data of the ozone content in the hydrate formed in each hydrate-forming experiment are summarized in Table S1. A counterpart to this set of "hydrate-side" data is given in Table S2; it is a set of "gas-phase-side" composition data obtained by sampling the gas mixture in the reactor at the end of each hydrate-forming process. It should be noted that, due to the insufficient reproducibility of the gas-exchange operations during each hydrate-forming experiment, the actual compositions measured with individual gas-mixture samples were erratically deviating from the corresponding *nominal* composition indicated on the leftmost column of the table. Because the hydrate formation had almost ceased at the time of such gas sampling in every hydrate-forming experiment, we can reasonably assume that the ozone content of each hydrate specified in Table S1 is nearly in a thermodynamic equilibrium relation to the corresponding gas-phase composition specified in Table S2.

The $x_{\mathrm{CO3,init}}$ and X_{O3} data given in Tables S1 and S2 were used to prepare Fig. 3 and 4 in the main body of this paper. If we replace X_{O3} on the abscissa in Fig. 4 with p_{O3}, the partial pressure of ozone in the gas phase, we have the $x_{\mathrm{CO3,init}}$ versus p_{O3} diagram shown in Fig. S1. Although the $x_{\mathrm{CO3,init}}$ data points for each level of the system pressure p seem to be correlated with p_{O3}, we note a systematic shift in the data points depending on p. Obviously, all the data points are better correlated with X_{O3} (see Fig. 4) rather than with p_{O3}.

Table S1. Summarized iodometric data of $x_{CO3,init}$, the mass fraction of ozone in the hydrate formed from an $O_3 + O_2 + CO_2$ gas mixture with a prescribed $(O_3 + O_2)$-to-CO_2 molar ratio under a nearly constant system pressure *p*. Each $x_{CO3,init}$ value listed here is an arithmetic mean of the values determined by three independent measurements. Note that the "O_3 + O_2 : CO_2" values indexed on the leftmost column represent the *nominal* feed-gas compositions, instead of the actually measured compositions.

$O_3 + O_2 : CO_2$	$x_{O3,init} \times 100$		
	p = 2.0 MPa	p = 2.5 MPa	p = 3.0 MPa
1 : 9	0.318	0.215	0.217
2 : 8	0.472	0.373	0.575
3 : 7	0.879, 0.640	0.715	0.695
4 : 6	0.791	0.783	0.912

Table S2. Summarized gas-chromatographic data of mole fractions of O_3, O_2 and CO_2 in the gas phase filling the reactor at the end of each hydrate-forming experiment.

$O_3 + O_2 : CO_2$	p / MPa	X_{O3}	X_{O2}	X_{CO2}
1 : 9	2.0	0.007	0.130	0.863
	2.5	0.006	0.079	0.915
	3.0	0.008	0.076	0.916
2 : 8	2.0	0.015	0.182	0.803
	2.5	0.010	0.171	0.819
	3.0	0.013	0.164	0.823
3 : 7	2.0	0.015	0.282	0.703
	2.0	0.013	0.331	0.657
	2.5	0.018	0.253	0.730
	3.0	0.018	0.273	0.709
4 : 6	2.0	0.017	0.393	0.590
	2.5	0.017	0.433	0.551
	3.0	0.021	0.409	0.570

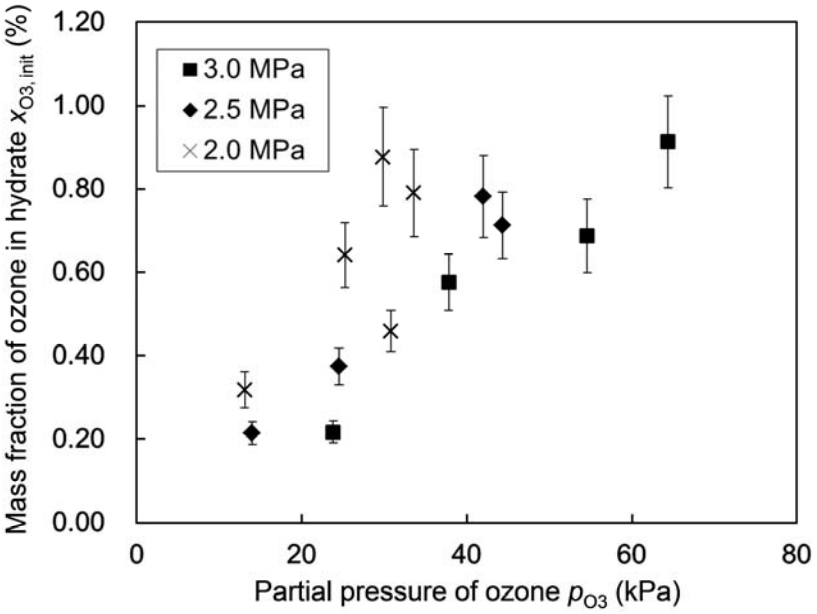

Figure S1. The initial ozone fraction (mass basis) in the formed hydrate, $x_{O3,init}$, versus p_{O3}, the partial pressure of ozone in the gas phase filling the reactor at the end of each hydrate-forming experiment. The legend inserted in the graph indicates the system pressure p during each hydrate-forming operation. The error bar for each data point represents the uncertainty of the ozone-fraction measurement by iodometry.
