# Supplementary material for: Molecular Storage of Ozone in a Clathrate Hydrate: An Attempt at Preserving Ozone at High Concentrations
Source: PLoS One. 2012 Nov 5;7(11):e48563. doi: 10.1371/journal.pone.0048563 (PMC3489668; doi:10.1371/journal.pone.0048563)
Supplement: Supporting Information S2 — This document contains Table S3 and Figure S2. (PDF) [file pone.0048563.s002.pdf]

Molecular Storage of Ozone in a Clathrate Hydrate: An Attempt at Preserving Ozone at High Concentrations

Takahiro Nakajima, Taisuke Kudo, Ryo Ohmura, Satoshi Takeya, and Yasuhiko H. Mori

---

Supporting Information S2:

Estimation of cage occupancies by ozone molecules

According to the well-known analogy to the Langmuir adsorption theory [S1], $\theta_{i,j}$, the occupancy (the fraction of occupation) of hydrate cages of type *j* by molecules of species *i*, can be related to f_i, the fugacity or, if an ideal-gas approximation is valid, partial pressure of species *i* in the gas phase in equilibrium with the hydrate, as follows:

$$\theta_{i,j} = \frac{C_{i,j} f_i}{1 + \sum_i C_{i,j} f_i} \quad , \qquad \text{(S1)}$$

where $C_{i,j}$ denotes the Langmuir constant for the filling of type *j* cages by molecules of species *i*. Applying Eq. (S1) to the system of present interest, we obtain the following six expressions for the cage occupancies relevant to this system:

$$\theta_{\mathrm{O3},s} = \frac{C_{\mathrm{O3},s} f_{\mathrm{O3}}}{1 + C_{\mathrm{O3},s} f_{\mathrm{O3}} + C_{\mathrm{O2},s} f_{\mathrm{O2}} + C_{\mathrm{CO2},s} f_{\mathrm{CO2}}} \quad , \qquad \text{(S2)}$$

$$\theta_{\mathrm{O3},l} = \frac{C_{\mathrm{O3},l} f_{\mathrm{O3}}}{1 + C_{\mathrm{O3},l} f_{\mathrm{O3}} + C_{\mathrm{O2},l} f_{\mathrm{O2}} + C_{\mathrm{CO2},l} f_{\mathrm{CO2}}} \quad , \qquad \text{(S3)}$$

$$\theta_{\mathrm{O2},s} = \frac{C_{\mathrm{O2},s} f_{\mathrm{O2}}}{1 + C_{\mathrm{O3},s} f_{\mathrm{O3}} + C_{\mathrm{O2},s} f_{\mathrm{O2}} + C_{\mathrm{CO2},s} f_{\mathrm{CO2}}} \quad , \qquad \text{(S4)}$$

$$\theta_{\mathrm{O2},l} = \frac{C_{\mathrm{O2},l} f_{\mathrm{O2}}}{1 + C_{\mathrm{O3},l} f_{\mathrm{O3}} + C_{\mathrm{O2},l} f_{\mathrm{O2}} + C_{\mathrm{CO2},l} f_{\mathrm{CO2}}} \quad , \qquad \text{(S5)}$$

$$\theta_{\mathrm{CO2},s} = \frac{C_{\mathrm{CO2},s}\, f_{\mathrm{CO2}}}{1 + C_{\mathrm{O3},s}\, f_{\mathrm{O3}} + C_{\mathrm{O2},s}\, f_{\mathrm{O2}} + C_{\mathrm{CO2},s}\, f_{\mathrm{CO2}}} \ , \tag{S6}$$

$$\theta_{\mathrm{CO2},l} = \frac{C_{\mathrm{CO2},l}\, f_{\mathrm{CO2}}}{1 + C_{\mathrm{O3},l}\, f_{\mathrm{O3}} + C_{\mathrm{O2},l}\, f_{\mathrm{O2}} + C_{\mathrm{CO2},l}\, f_{\mathrm{CO2}}} \ , \tag{S7}$$

where subscripts s and l denote the small (5^{12}) and large ($5^{12}6^{2}$) cages, respectively. The Langmuir constants for O_2 and CO_2 can be derived from the relevant Kihara parameters reported by Mehta and Sloan [S2] and Herri et al. [S3], respectively, as follows:

$$C_{\mathrm{O2},s} = 7.41 \times 10^{-7} ,\ C_{\mathrm{O2},l} = 3.88 \times 10^{-6} , \tag{S8}$$

$$C_{\mathrm{CO2},s} = 3.81 \times 10^{-6} ,\ C_{\mathrm{CO2},l} = 5.82 \times 10^{-5} . \tag{S9}$$

The fugacities of O_2 and CO_2 can be obtained from an NIST database [S4]. The fugacity of O_3 may be approximated by its partial pressure, p_{O3}, with a reasonable accuracy. Thus, the yet-to-be determined quantities included on the right-hand side of each of the six cage-occupancy epressions, Eqs. (S2)–(S7), are $C_{\mathrm{O3},s}$ and $C_{\mathrm{O3},l}$, the Langmuir constants for O_3. These two quantities can be determined by a regression analysis, which is outlined below.

The mass fraction of O_3 in the hydrate, x_{O3}, can be expressed in terms of the six cage occupancies, $\theta_{i,j}$, and the molar masses, M_i, of the four hydrate-constituting substances:

$$x_{\mathrm{O3}} = \frac{(2\,\theta_{\mathrm{O3},s} + 6\,\theta_{\mathrm{O3},l})M_{\mathrm{O3}}}{46\,M_{\mathrm{H2O}} + (2\,\theta_{\mathrm{O3},s} + 6\,\theta_{\mathrm{O3},l})M_{\mathrm{O3}} + (2\,\theta_{\mathrm{O2},s} + 6\,\theta_{\mathrm{O2},l})M_{\mathrm{O2}} + (2\,\theta_{\mathrm{CO2},s} + 6\,\theta_{\mathrm{CO2},l})M_{\mathrm{CO2}}} \ , \tag{S10}$$

where M_i / [kg mol^{-1}] = 47.9982 for O_3, 31.9988 for O_2, 44.0095 for CO_2, and 18.0153 for H_2O [S5]. A regression analysis was completed to determine the $C_{\mathrm{O3},s}$ and $C_{\mathrm{O3},l}$ values that minimize the sum of the squares of the deviations of the x_{O3} values calculated by Eq. (S10) from the corresponding $x_{\mathrm{O3,init}}$ values experimentally determined. Following are the $C_{\mathrm{O3},s}$ and $C_{\mathrm{O3},l}$ values thus determined:

$$C_{\mathrm{O3},s} = 1.48 \times 10^{-5} ,\ C_{\mathrm{O3},l} = 6.58 \times 10^{-6} . \tag{S11}$$

Substituting the $C_{i,j}$ values given in Eqs. (S8), (S9) and (S11) into Eqs. (S2)–(S7) together with the f_i values corresponding to each of the experimental gas-phase conditions given in Table S2, we determined the $\theta_{i,j}$ values as summarized in Table S3. The $\theta_{\mathrm{O3},s}$ and $\theta_{\mathrm{O3},l}$ values are plotted versus p_{O3}, the partial pressure of ozone in the gas phase, in Figure S2.

Table S3. Cage occupancies estimated from the gas-phase composition data summarized in Table S2.

$O_3 + O_2 : CO_2$	p / MPa	$\theta_{O3,s}$	$\theta_{O3,l}$	$\theta_{O2,s}$	$\theta_{O2,l}$	$\theta_{CO2,s}$	$\theta_{CO2,l}$
1 : 9	2.0	0.0267	0.0009	0.0266	0.0110	0.8081	0.9771
	2.5	0.0236	0.0008	0.0168	0.0067	0.8455	0.9838
	3.0	0.0348	0.0012	0.0167	0.0066	0.8496	0.9847
2 : 8	2.0	0.0632	0.0024	0.0373	0.0163	0.7605	0.9697
	2.5	0.0429	0.0015	0.0375	0.0156	0.8010	0.9734
	3.0	0.0569	0.0020	0.0371	0.0154	0.8040	0.9745
3 : 7	2.0	0.0657	0.0025	0.0619	0.0280	0.7236	0.9566
	2.0	0.0579	0.0023	0.0757	0.0347	0.7110	0.9495
	2.5	0.0795	0.0030	0.0565	0.0251	0.7426	0.9616
	3.0	0.0860	0.0032	0.0642	0.0284	0.7432	0.9593
4 : 6	2.0	0.0798	0.0033	0.0929	0.0448	0.6667	0.9372
	2.5	0.0864	0.0035	0.1104	0.0530	0.6641	0.9307
	3.0	0.1102	0.0045	0.1041	0.0498	0.6698	0.9351

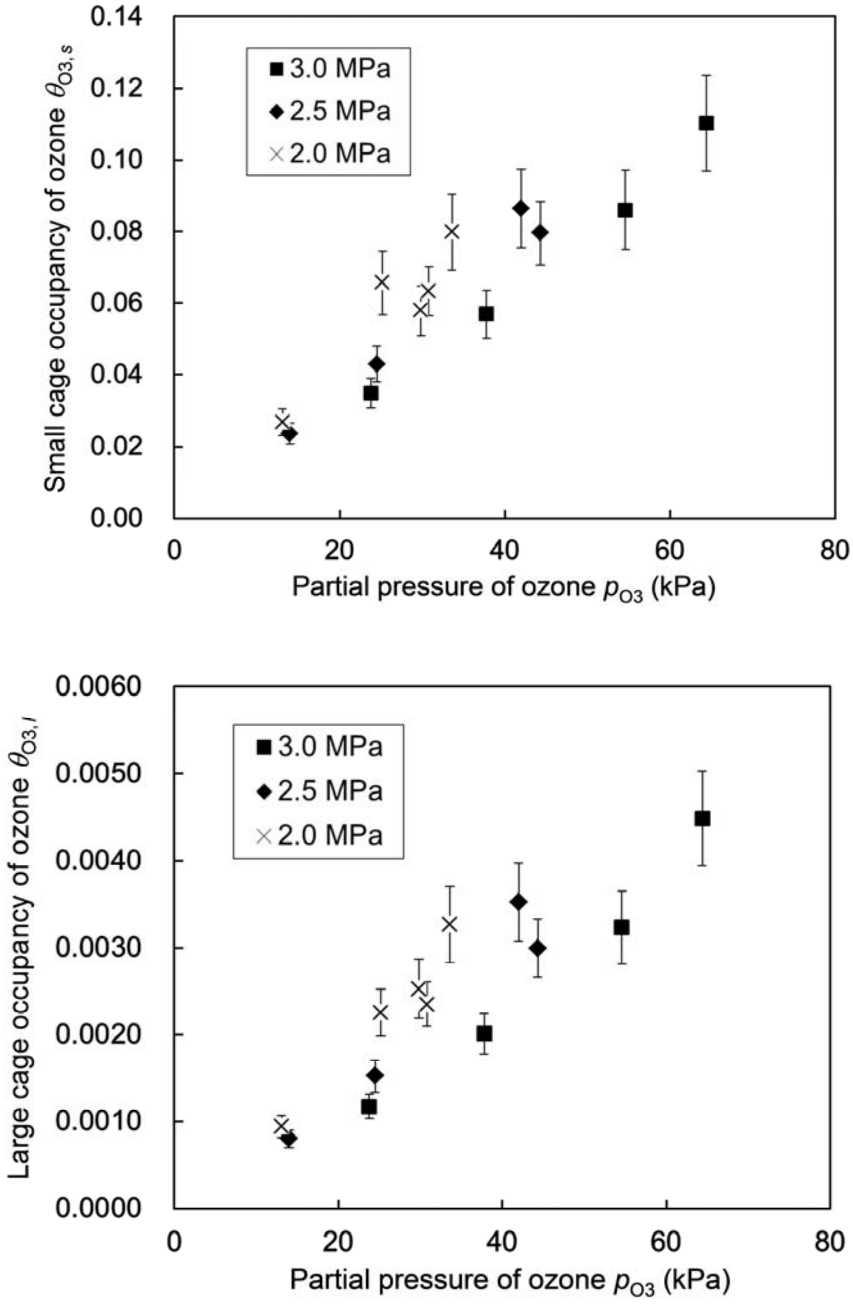

Figure S2. Occupancies of small (5^{12}) and large ($5^{12}6^{2}$) cages of an sI hydrate by ozone molecules versus partial pressure of ozone in the gas phase in contact with the hydrate. The legend inserted in the graph indicates the system pressure *p* during each hydrate-forming operation. The error bar for each data point represents the uncertainty of the ozone-fraction measurement by iodometry.

References

[S1] Sloan ED, Koh CA (2008) Clathrate Hydrates of Natural Gases, 3rd ed. Boca Raton: CRC Press. p. 266.

[S2] Mehta AP, Sloan ED (1996) Improved thermodynamic parameters for prediction of structure H hydrate equilibria. AIChE J 42: 2036–2046.

[S3] Herri JM, Bouchemoua A, Kwaterski M, Fezoua A, Ouabbas Y, Cameirao A (2011) Fluid Phase Equilib 301: 171–190. The Kihara parameters relevant to “Model 1” (indicated in Table 5 of this paper) were used for deriving Eq. (S9).

[S4] Lemmon E, Huber M, McLinden M (2007) NIST Reference Fluid Thermodynamic and Transport Properties Database (REFPROP), Ver. 8.0. Gaithersburg, MD: US Department of Commerce.

[S5] NIST Chemistry WebBook. Available: http://webbook.nist.gov/chemistry/. Accessed 10 July 2012.
